# The protective capacity of high payload FMDV A22 IRQ vaccine in sheep against direct-contact challenge with a heterologous, contemporary FMDV A strain from South East Asia

**DOI:** 10.1371/journal.pone.0195302

**Published:** 2018-06-18

**Authors:** Jacquelyn Horsington, Charles Nfon, Hilary Bittner, Peter A. Durr, Nagendrakumar Singanallur, Soren Alexandersen, Wilna Vosloo

**Affiliations:** 1 Australian Animal Health Laboratory, CSIRO, Geelong, Victoria, Australia; 2 National Centres for Animal Disease, Canadian Food Inspection Agency, Winnipeg, Manitoba, Canada; 3 Geelong Centre for Emerging Infectious Diseases, Geelong, Victoria, Australia; 4 Deakin University Geelong, Geelong, Victoria, Australia; 5 Barwon Health, University Hospital Geelong, Geelong, Victoria, Australia; Pirbright Institute, UNITED KINGDOM

## Abstract

Foot-and-mouth disease (FMD) is an acute, highly contagious viral disease of domestic and wild cloven-hoofed animals, caused by FMD virus (FMDV). An FMD outbreak can cause major production losses and have significant implications for trade. Vaccination can assist in controlling the disease, and emergency vaccination using high antigen payload vaccines (>6 PD_50_/dose) is considered an important control approach in the event of an outbreak. In recent years there has been a divergence of serotype A viruses in South East Asia (SEA) into several distinct genetic and antigenic clusters. Numerous variants were found to poorly match serotype A vaccines commonly included in international antigen banks. This study examined the ability of single vaccination with high-potency monovalent A22 IRQ vaccine to protect sheep following challenge with the A/VIT/15/2012 strain, just four days following vaccination. The vaccine proved effective at limiting clinical disease but did not prevent infection.

## Introduction

Foot-and-mouth disease (FMD) is a highly infectious disease that affects ruminants and pigs, caused by FMD virus (FMDV), a small, positive-sense RNA virus in the Genus *Aphthovirus*, Family *Picornaviridae*. Cattle, pigs, sheep and goats are the livestock species that play an important role in the epidemiology of FMD in many parts of the world, and sheep have been central to the spread of infection in numerous outbreaks [1, 2]. This spread is facilitated by cases often going undetected, as clinical signs of FMD in sheep are frequently mild or inapparent. Sheep, like other ruminants, can also become persistently infected with FMDV [3, 4].

Vaccination is often used to assist in controlling the disease, however, there is little or no cross-protection between the seven different serotypes, and varied cross-protection between different strains of the same serotype [5–8]. Emergency vaccination using high antigen payload vaccines (>6 protective dose (PD)_50_/dose) is considered an important control approach in the event of an outbreak in some FMD-free countries, reducing the extent of FMDV excretion, limiting the possibility of transmission and potentially minimising the duration and intensity of an outbreak. Previous studies have shown high-potency vaccination to be effective at protecting animals challenged as early as four days post-vaccination (dpv) [9–13].

Serotype A is one of the most genetically and antigenically diverse of the FMDV serotypes and new antigenic variants emerge frequently [14, 15]. Over the last ten years there has been a divergence of serotype A viruses in South East Asia (SEA) into several distinct genetic and antigenic clusters. Variants from Vietnam in 2012 were found to match poorly (poor r1 values in *in vitro* vaccine matching studies) with the serotype A vaccines commonly included in international antigen banks (WRL, Pirbright).

The objective of this study was to determine if a single vaccination with an emergency monovalent A22 IRQ vaccine with high antigen payload (>6 PD_50_/dose) can confer protection in sheep and prevent the development of persistent infection following direct-contact challenge with the heterologous A/VIT/15/2012 strain, which belongs to the SEA97 lineage, in an emergency vaccination scenario. To reflect an outbreak situation, where the time between vaccination and challenge may be minimal, vaccinated animals were challenged just four days following vaccination. The vaccine proved effective at limiting clinical disease, but did not prevent infection.

## Materials and methods

### Ethics statement

This study was approved by the Canadian Centre for Human and Animal Health Animal Care Committee (AUD# C-14-002) and Australian Animal Health Laboratory’s Animal Ethics Committee (AEC 1680) and performed in strict accordance with the recommendations in the Canadian Council for Animal Care Guidelines and the Australian Code of Practice for the Care and Use of Animals for Scientific Purposes.

### Animals

Thirty Rideau Arcott/Ile de France rams aged between 6 and 12 months (approximately 40 kg) were used. All animals were housed in the BSL3 animal facility at the National Centre for Foreign Animal Disease (NCFAD), Winnipeg, Canada. The sheep were divided into three groups: unvaccinated, coronary band (CB) inoculated donor sheep (n = 18); vaccinated contact-challenged (VC) sheep (n = 6); and unvaccinated contact-challenged (UC) sheep (n = 6).

### Vaccination

The VC sheep were vaccinated with one full sheep dose (1 ml) of high antigen payload (>6 PD_50_/dose) FMDV A22 IRQ double-oil emulsion vaccine, formulated by Merial Animal Health, Pirbright, UK, and administered intramuscularly in the neck region above the left shoulder. Vaccination was given 4 days prior to challenge.

### Challenge virus

The challenge virus, A/VIT/15/2012, was isolated from pigs in Vietnam in 2012 and belongs to the Asia topotype (SEA-97 lineage). After original isolation on baby hamster kidney cells, the virus was passaged through cattle. Inoculum prepared from lesion material was titrated in two cattle by tongue titration and the cattle infectious dose (CID_50_) was calculated to be ~10^8^/ml. The r1 value in a virus neutralisation test (VNT) vaccine matching assay was 0.17 against A22 IRQ (WRL, Pirbright).

### Experimental design

Eighteen donor sheep were each inoculated intradermally into the CB with 5 log_10_ CID_50_ of virus in a volume of 0.1 ml. The VC and UC sheep were challenged by continuous direct contact with the donor sheep, starting 4 hrs after their inoculation (designated as 0 days post-challenge (dpc)). The sheep were arranged into groups of five, housed in six separate rooms, with one VC sheep, one UC sheep and three directly inoculated donor sheep per room. The sheep distribution per room is described in Table 1.

**Table 1:** Allocation of sheep per room and day post-challenge each animal was euthanized.

Room	Sheep No.	Challenge	Vaccinated	Day Euthanized
1	7	Contact	Yes	9
	13	Contact	No	9
	19	CB	No	9
	20	CB	No	9
	21	CB	No	9
2	8	Contact	Yes	35
	14	Contact	No	10
	22	CB	No	9
	23	CB	No	9
	24	CB	No	9
3	9	Contact	Yes	35
	15	Contact	No	12
	25	CB	No	9
	26	CB	No	9
	27	CB	No	9
4	10	Contact	Yes	35
	16	Contact	No	21
	28	CB	No	9
	29	CB	No	9
	30	CB	No	9
5	11	Contact	Yes	35
	17	Contact	No	21
	31	CB	No	8
	32	CB	No	9
	33	CB	No	9
6	12	Contact	Yes	35
	18	Contact	No	12
	34	CB	No	9
	35	CB	No	9
	36	CB	No	9

### Monitoring and sample collection

The sheep were monitored for the development of clinical signs such as pyrexia, lameness and development of vesicles with rectal temperatures and clinical scores recorded daily to 14 dpc. Sheep showing elevated temperatures (>40.5°C) were considered as having pyrexia. Clinical scores were calculated as follows: per foot, a score of 1 if a lesion developed in one location (CB, interdigital cleft or heel pad) and a score of 2 if lesions developed in two or more locations; 1 each for oral (tongue, gums or dental pad) lesions, 1 if visibly lame/slow to rise. The maximum score was 10.

Clinical samples were collected from the donor and in-contact animals at regular intervals for 35 days. Blood in EDTA tubes for RT-qPCR and clotted blood for serology were collected at -4 dpc, daily between 0 and 14 dpc and then weekly to 35 dpc. Small sterile cotton buds were used to collect nasal and saliva secretions at the same time points. Rectal swabs were collected daily between 0 and 4 dpc. All swabbing was performed in duplicate and swabs placed in tubes containing 500 μl of phosphate buffered saline (PBS) for RT-qPCR or 500 μl of Dulbecco’s modified Eagle’s medium (DMEM) containing 0.04 M HEPES and antibiotics (catalog no. 15240062; Gibco) for virus isolation. Oro-pharyngeal fluid (OPF) was collected at -1, 7, 9, 14, 21, 28 and 35 dpc, using a small probang sampling cup and mixed with 2 ml DMEM containing 0.04 M HEPES. For animals that were euthanized on day 12, samples were collected at this time point instead of day 14. At the time of sacrifice, sheep were sedated with 2% Xylazine at 0.67 ml/100 Kg body weight and euthanized by intravenous injection of sodium pentobarbital (100mg/kg IV).

### Virus isolation

Oro-pharyngeal fluid samples were examined for the presence of live virus by inoculation onto αVβ6-expressing porcine kidney (LFBK-αVβ6) cells [16, 17] grown in 24-well cell culture trays, and incubated for 30 mins at 37°C. The cells were washed with PBS and overlayed with DMEM containing 5% foetal bovine serum and antibiotics (catalog no. 15240062; Gibco), then examined for cytopathic effect after 24 and 48 hrs incubation at 37°C with 5% CO_2_. If no cytopathic effect was observed, a blind passage followed. All supernatants from positive wells were tested using an FMDV antigen enzyme-linked immunosorbent assay (ELISA) and A22 IRQ reagents [18].

### Detection of FMDV RNA by RT-qPCR

The amount of viral RNA in whole blood, OPF and nasal, oral and rectal swab samples was quantified by a TaqMan RT-qPCR assay as described previously [11]. Samples with a Ct >40 (equivalent to 1 x 10^3.5^ copies RNA/ml blood or 1 x 10^3.2^ copies RNA/swab) were considered negative.

### Determination of neutralising antibody titre

Heat inactivated (56°C, 30 min) serum samples were used for VNT on swine kidney (IBRS2) cells using A/VIT/15/2012 and A22 IRQ viruses previously adapted to IBRS2 cells by passaging 5 times. Titres ≥1.2 log_10_ (1:16) were considered positive (OIE Manual of Diagnostic Tests and Vaccines for Terrestrial Animals).

### Detection of antibodies to non-structural and structural proteins by ELISA

Sera were tested for the presence of antibodies against viral non-structural proteins (NSP) by an NCFAD in-house competitive ELISA (3ABC-ELISA) [18] and for the presence of antibodies against FMDV type A structural proteins (SP) by A-serotype specific competitive ELISA (cELISA) using reagents homologous to A22 IRQ and a protocol similar to that described by Mackay [19].

### Statistical analysis

The total viral excretion of each donor was estimated by calculating the sum of the integral of the curve of its daily oral and nasal viral load, as estimated by the RT-qPCR. Estimates were calculated for the first 6 days of the experiment, as this is noted in the literature as the time sheep are excreting most virus [20]. The total viral load for this time period for each room used in the infection study was graphed using boxplots, and an assessment of whether or not there were significant differences between the rooms was tested using the Kruskal-Wallis non parametric One-Way ANOVA by Ranks test.

As an estimate of the capacity of vaccination to prevent overt clinical disease we used McNemar’s test on the 2 x 2 table of vaccination and disease status, with animals being matched by their room and any animal with a score ≥1 being classified as overtly diseased, or else a classification of not overtly diseased.

To determine the median time for seroconversion of the donor and two in-contact groups of sheep we used a Kaplan-Meier non-parametric time-to-event analysis. Right censoring was enabled to allow for sheep that were euthanized before seroconversion had occurred. The start time for the estimates was the day of inoculation/contact. To test for significant differences in the time to seroconversion for the vaccinated and non-vaccinated in-contact sheep, we used a log-rank test.

All analyses were done using *R* version 3.2.4 (https://www.r-project.org/). To determine the integral of the daily viral load plots we used the “*trapz*” function of the “*pracma*” library. For the Kruskal-Wallis test we used the “*kruskal*.*test*” function, for the McNemar’s test we used “*mcnemar*.*test*” (with the continuity correction being applied). All of these functions belong to the core *R* “*stats”* library. For the calculation of the median times of sero-conversions and for the log-rank test, the “*survfit*” and “*survdiff*” functions from the “*survival*” library. Comparison of virus excretion between the vaccinated and unvaccinated contacts were performed using ANOVA with Single Factor and Fisher’s Exact Test.

## Results

### Donor sheep

#### Clinical signs and viraemia

This study comprised six replicate rooms with three donor sheep and one VC and one UC sheep per room (Table 1). The clinical scores and FMDV RNA load in blood, nasal swabs and oral swabs for each donor sheep are shown in S1 Fig. All CB inoculated donor animals developed generalised FMD with multiple lesions on the feet, mouth and tongue as early as 2 days post-challenge (dpc). On average, the rectal temperatures of the sheep in the donor group were elevated with pyrexia recorded in 14 of the 18 sheep between 1 to 9 dpc. Sheep 31 developed severe clinical disease with extensive lesions on all feet and was euthanized for ethical reasons at 8 dpc. At 9 dpc, severe clinical signs including necrosis and hoof sloughing were also observed in sheep 24, 32 and 36. As these sheep required euthanasia, the decision was made to cull all donors on this day to retain equivalence between the rooms.

No viraemia was detected in sheep 20 or 29. In most other donor sheep viraemia lasted three or four days (S1 Fig). However, for sheep 23 and 31 viraemia persisted for five days. Virus was isolated on at least one day from the blood of all animals that were positive in RT-qPCR, except sheep 28 and 35.

#### Virus in nasal and oral secretions

Nasal and oral swab samples from most donor sheep were positive for FMDV RNA from as early as 1 dpc, and in some animals up to 9 dpc, with viral loads generally decreasing from 4–6 dpc (S1 Fig). Most animals were positive in nasal swab samples for four or more consecutive days, and in oral swab samples for three or more consecutive days. Sheep 28 had only one positive oral swab sample at 5 dpc, and all samples from sheep 29 were negative. Rectal swabs from all sheep were negative on all days tested.

**Fig 1:**
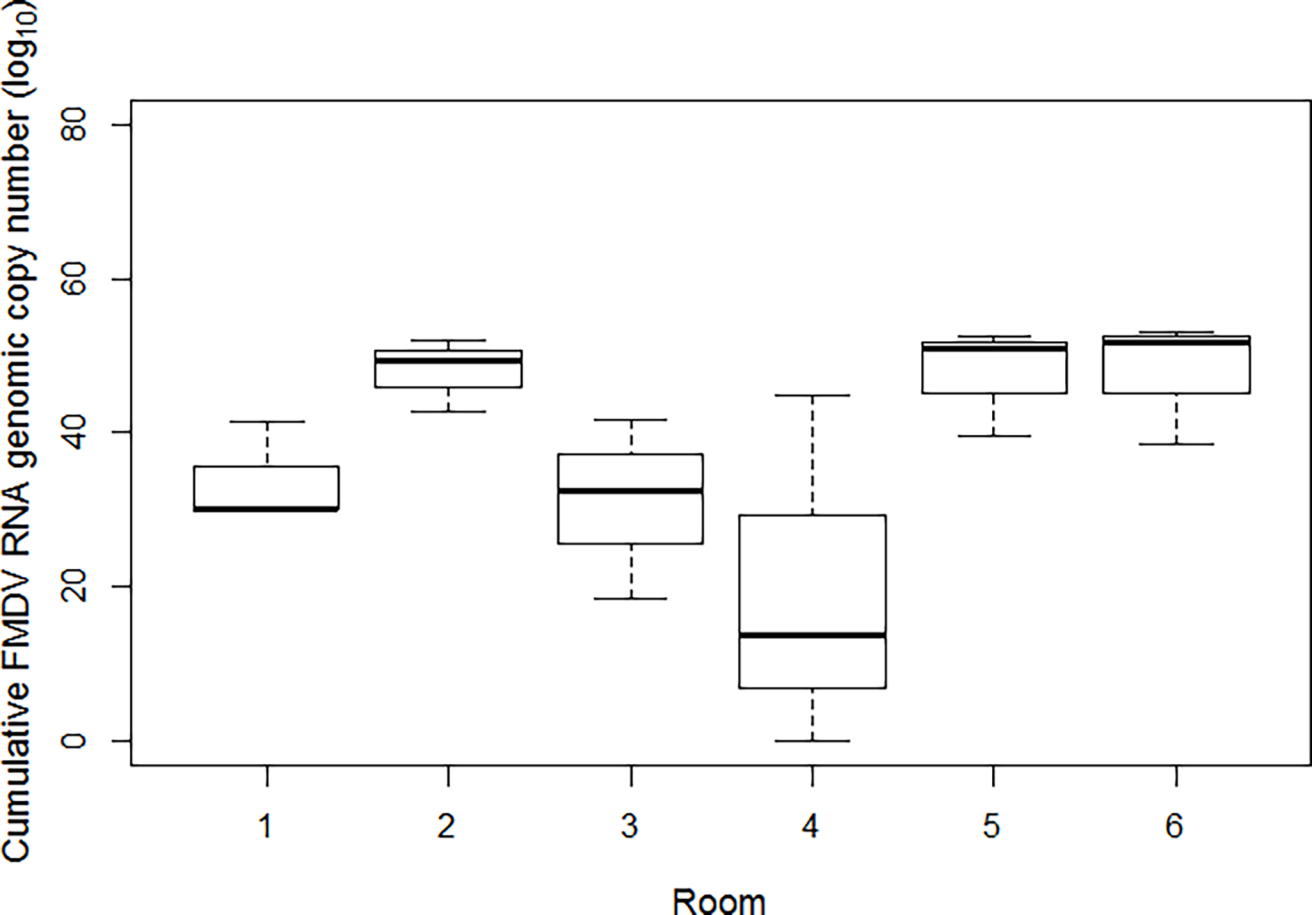
Box plot of accumulated viral RNA loads in NS and OS from the three donor sheep per room over the first 6 dpc.

The amount of virus detected in nasal and oral secretions can reflect the amount of virus excreted, and subsequently the level of challenge to the in-contact sheep. The Kruskal-Wallis analysis of total viral load in nasal and oral swabs from each group of three donor sheep indicated no significant difference between the rooms over the first 6 dpc (p = 0.1148; Fig 1).

Oropharyngeal fluid samples from all donor sheep were positive for FMD virus and/or viral RNA at 7 and/or 9 dpc (when all donors were euthanized), with the exception of sheep 20 and 29 (data not shown).

#### Immune response

**Fig 2:**
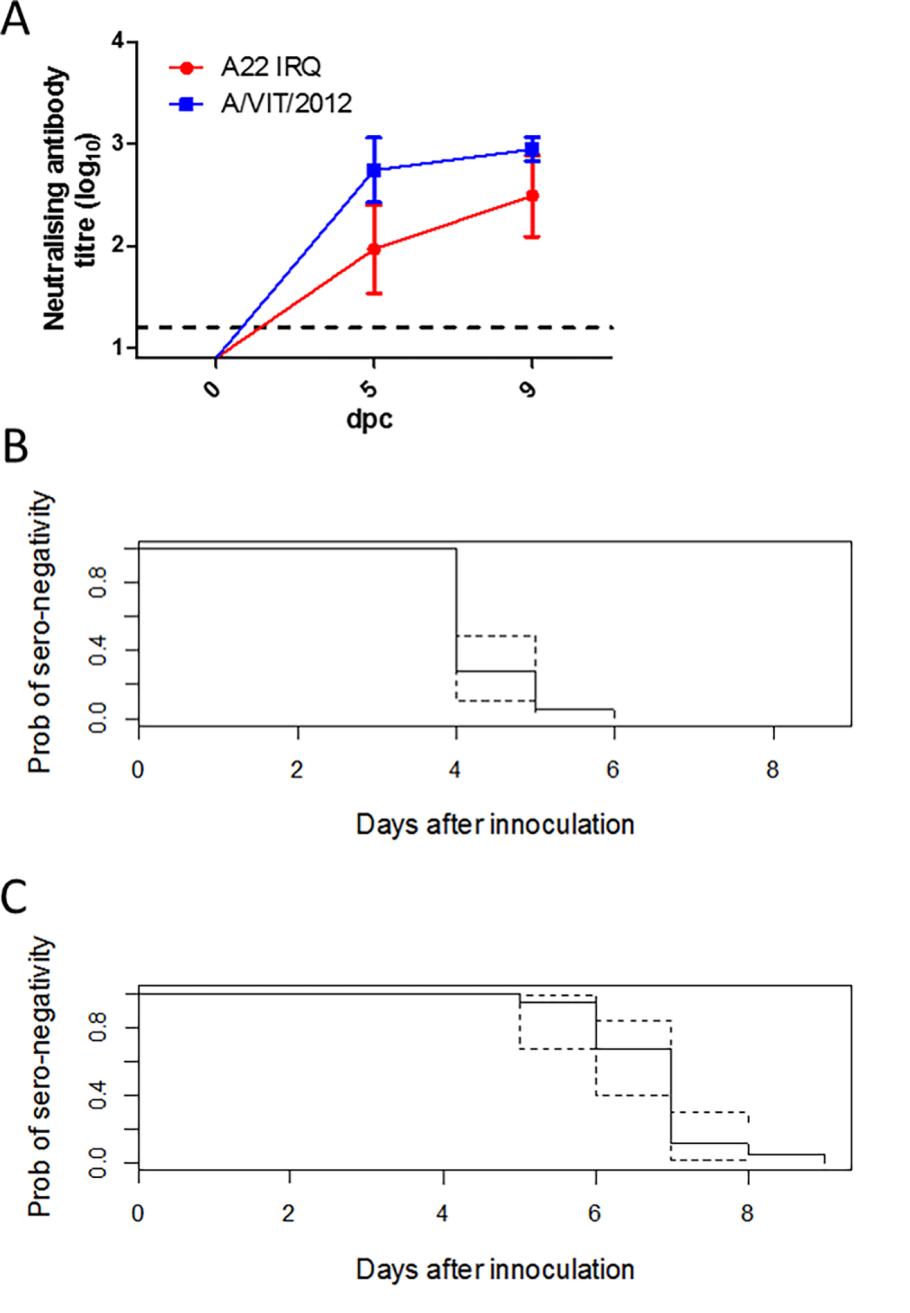
(A) Average neutralising antibody titres of the donor sheep sera to the vaccine virus (A22 IRQ) and the challenge virus (A/VIT/2012). Values represent Log_10_ of the reciprocal of the highest dilution of serum that was able to neutralise either 100 TCID_50_ of A22 IRQ or A/VIT/2012. The assay cut off was 0.9, titres ≥1.2 are considered positive. Time to seroconversion plots estimated by the Kaplan-Meier method for the donor sheep (B) for the structural protein ELISA; and (C) for the non-structural protein ELISA. The dotted lines are the estimated 95% confidence limits.

All of the donors seroconverted to the challenge virus (A/VIT/15/2012) by 5 dpc (Fig 2A). The neutralising antibody titres to the vaccine strain virus (A22 IRQ) were lower overall compared to the challenge virus (A/VIT/15/2012), with one sheep negative at 5 dpc. The higher VN titres to the challenge strain compared to the vaccine strain reflects the r1 values. In ELISA, the median time to seroconversion, as assessed by the Kaplan-Meier time to event analysis, was 4 days (95% CL: 4, 4) for the structural proteins (Fig 2B) and 7 days (95% CL: 6, 7) for the NSP (Fig 2C).

### Contact sheep

#### Clinical signs and viraemia

**Fig 3:**
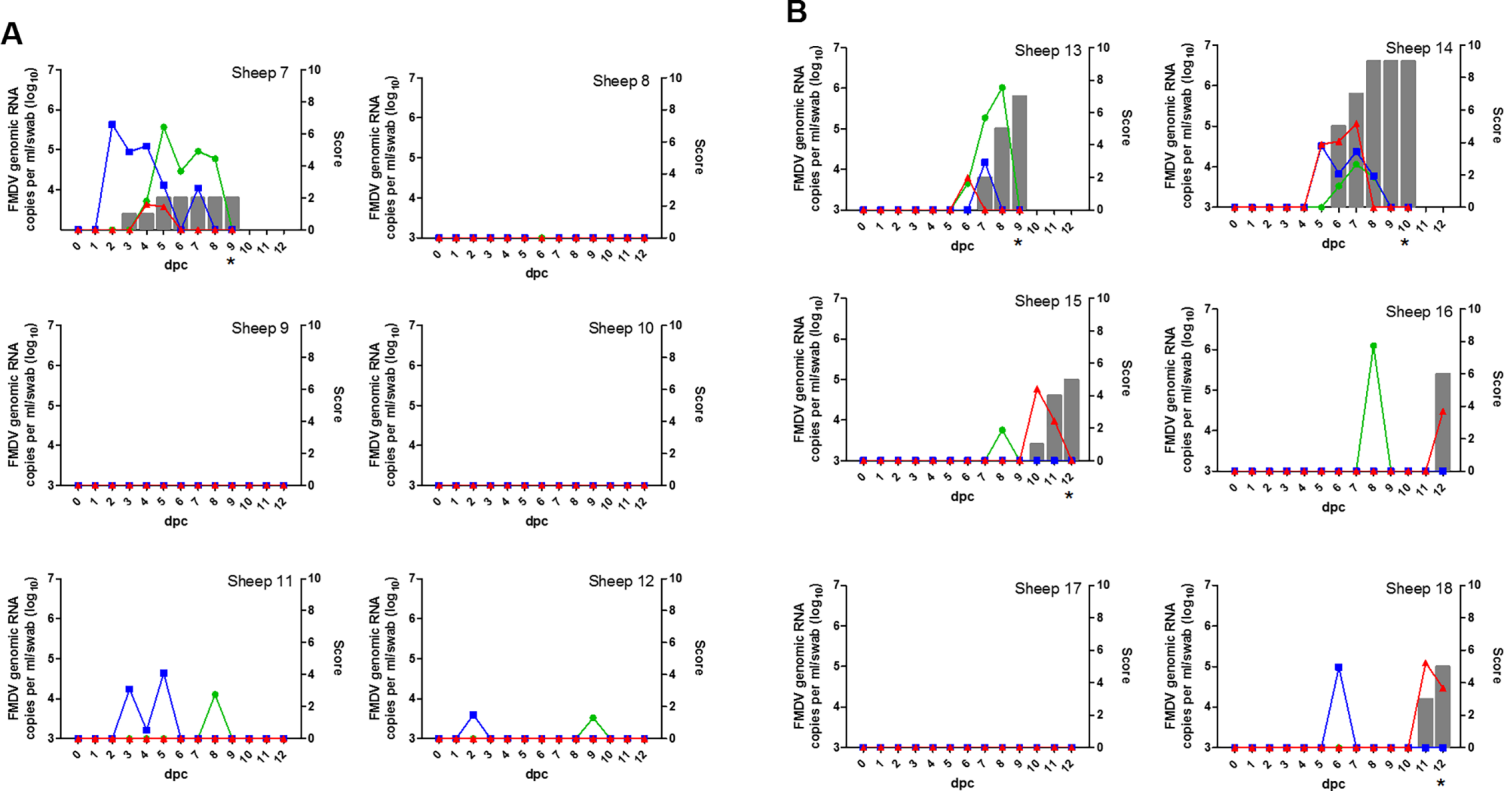
Disease dynamics in VC (A) and UC (B) sheep. FMDV RNA detection in blood (red), nasal (green) and oral (blue) swabs was performed using RT-qPCR, and is presented as log_10_ genome copy numbers/ml blood or swab. Clinical score is a cumulative index of FMD lesion distribution and clinical signs, where the maximum score is 10. * = animal euthanized.

Five of six VC sheep were protected from clinical disease (Fig 3A). Only one (sheep 7) developed systemic FMD with a lesion on the right-hind heel pad from 3 dpc. An interdigital lesion developed on the same foot by 5 dpc, followed by signs of hoof sloughing at 9 dpc. Consequently this animal was euthanized for ethical reasons. The majority of the VC sheep were not pyrexic with only sheep 7 having a rectal temperature above 40.5°C at 5 dpc.

Five of the six UC sheep developed systemic FMD (Fig 3B) with lesions on the feet and gums appearing between 6 and 12 dpc. Sheep 13 was euthanized on day 9 in line with ethical requirements for companionship, as the other sheep in this room had reached ethical endpoint. Sheep 14 developed severe disease on both hind feet and was euthanized on day 10. At 12 dpc, sheep 15 and 18 were euthanized for ethical reasons, followed by sheep 16 at 21 dpc. The final UC sheep (sheep 17) was also euthanized on this day to retain equivalence, however, no lesions were observed on necropsy. Rectal temperatures above 40.5°C were recorded for sheep 13–16 between 7 and 12 dpc, and for sheep 18 on 11 dpc.

Only one VC sheep (sheep 7, the same sheep that had lesions) was viraemic, with FMDV RNA detected in the blood at 4 and 5 dpc (Fig 3A). However, virus was not isolated from any of these RNA positive blood samples, despite RNA levels being comparable to that observed in the donor sheep. This is likely due to the presence of neutralising antibodies from vaccination. FMDV RNA was detected in the blood of five of the six UC sheep between 5 and 12 dpc (Fig 3B), and virus was isolated from sheep 14 at 5 dpc and 16 at 12 dpc. No viraemia was detected in sheep 17 at any time point.

Analysis of the presence of clinical disease (or not) for the room-paired sheep using McNemar’s test showed no significant difference between the VC and UC animals (p = 0.134).

#### Virus in nasal and oral secretions

FMDV RNA was detected in nasal swabs from sheep 7 from 5 to 8 dpc and in oral swabs between 2 and 7 dpc (Fig 3A). Sheep 11 and 12 were also positive for FMDV RNA in nasal and oral swab samples, however only on one or two sampling days. All other nasal and oral swab samples from the vaccinated sheep were negative.

Nasal swab samples from UC sheep 13–16 were FMDV RNA positive on at least one day between 6 and 10 dpc, and oral swab samples from three UC sheep (13, 14 and 18) were positive on at least one day between 5 and 9 dpc. Sheep 17 was negative in all samples from all days (Fig 3B).

Analysis of virus RNA levels in the nasal and oral secretions between groups indicated no significant difference between VC and UC sheep (oral secretions: p = 0.24 and p = 0.30; and nasal secretions: p = 0.54 and 0.62, by ANOVA with Single Factor and Fisher’s Exact Test, respectively).

Oropharyngeal fluid samples from five VC sheep were FMDV RNA positive during the acute phase of infection (7–9 dpc) and one of these, sheep 12, was positive in all samples to 35 dpc. Virus was isolated from the same five sheep between 7 and 14 dpc and from sheep 11 and 12 at 21, 28 and 35 dpc, suggesting these two animals were persistently infected (Table 2). The OPF from sheep 9 was not FMDV positive at any stage.

**Table 2:** Detection of FMDV in probang samples from vaccinated in-contact transmission sheep (VC) and unvaccinated in-contact transmission sheep (UC). Reported as detection by VI/RT-qPCR.

Group	Sheep No.	Days Post-challenge						
		-4	7	9	12*/14	21	28	35
VC	7	-/-	+/-	+/5.1^a^	nd	nd	nd	nd
	8	-/-	-/-	-/-	+/4.9	-/-	-/-	-/-
	9	-/-	-/-	-/-	-/-	-/-	-/-	-/-
	10	-/-	+/5.2	-/-	-/-	-/-	-/-	-/-
	11	-/-	+/6.8	+/10.5	-/-	+/3.4	+/-	+/3.3
	12	-/-	+/5.5	+/4.3	+/4.2	+/4.0	+/4.0	+/4.0
UC	13	-/-	+/4.3	-/4.1	nd	nd	nd	nd
	14	-/-	+/-	+/4.4	nd	nd	nd	nd
	15	-/-	-/-	-/-	+/4.9	nd	nd	nd
	16	-/-	-/-	+/-	+/-	nd	nd	nd
	17	-/-	-/-	-/-	-/-	nd	nd	nd
	18	-/-	+/-	-/-	-/3.4	nd	nd	nd

+ = VI positive; - = VI or RT-qPCR negative; nd–not done

^a^ log_10_ genome copy numbers/ml

*sheep 15 and 18 were sampled on day 12 not 14

FMDV and/or FMDV RNA was detected in the OPF samples from five UC sheep between 7 and 14 dpc (Table 2). All samples from sheep 17 were negative.

#### Immune response

**Fig 4:**
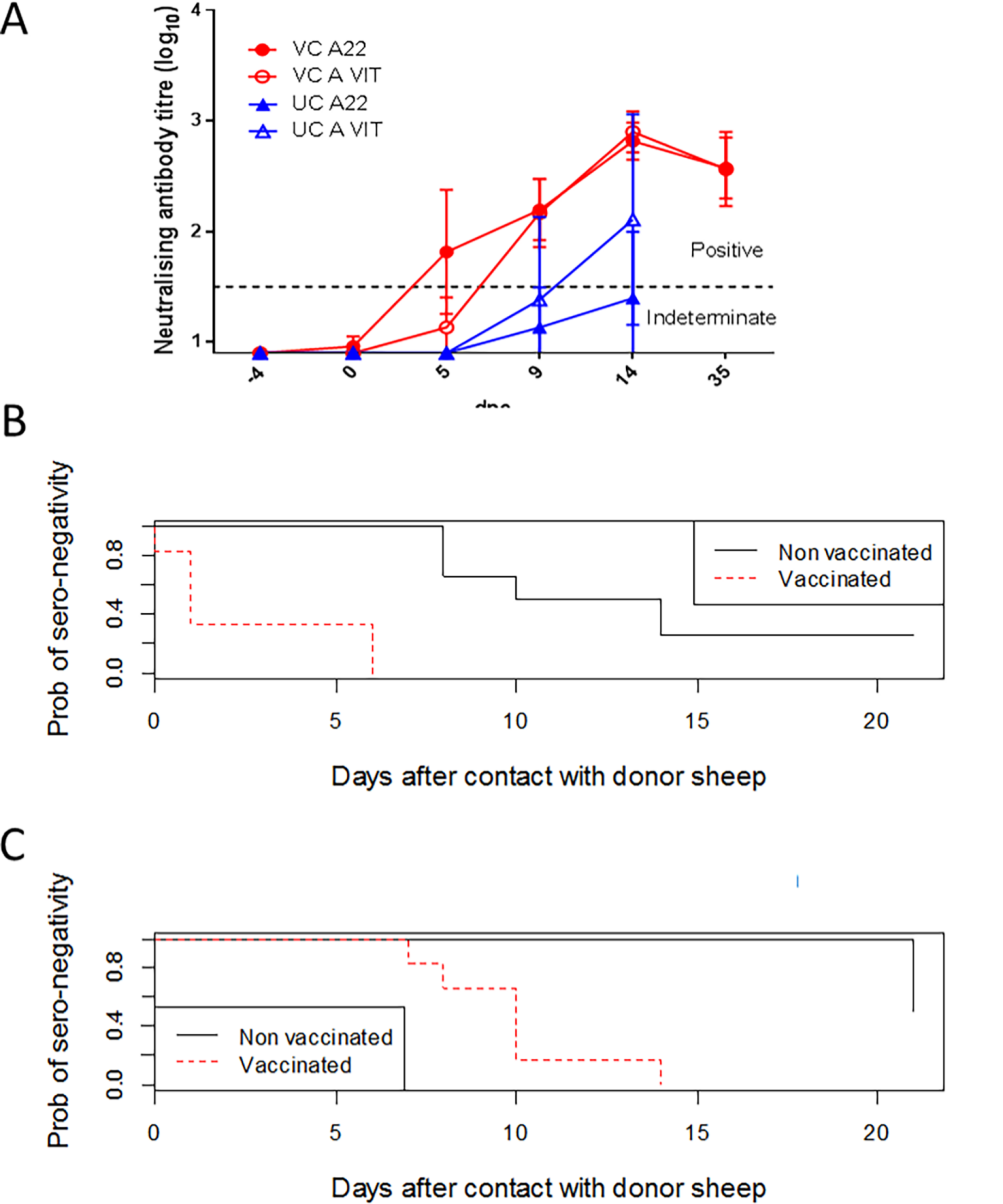
(A) Average neutralising antibody titres of the vaccinated (VC) and unvaccinated (UC) contact sheep to the vaccine virus (A22 Iraq) and the challenge virus (A/VIT/2012). Values represent Log_10_ of the reciprocal of the highest dilution of serum that was able to neutralise either A22 Iraq or A/VIT/2012. The assay cut off was 0.9, titres ≥1.2 are considered positive. Error bars show standard deviation. Time to seroconversion plots estimated by the Kaplan-Meier method for the vaccinated and non-vaccinated in-contact sheep (B) for the ELISA to the structural protein; and (C) for the ELISA to the non-structural protein. Note that for both groups of sheep, the start time for the estimate is when the sheep entered the room housing the donors, and not the day that the vaccine was administered.

At the time of challenge, two VC sheep had detectable neutralising antibodies to FMDV A22 IRQ, though at levels considered negative (titre of 1.08). The remaining VC sheep had no detectable neutralising antibodies, and all were negative for neutralising antibodies to FMDV A/VIT/15/2012 (Fig 4A). Five VC sheep seroconverted to FMDV A22 IRQ with neutralising antibodies by 5 dpc and the remaining sheep by 9 dpc, however against the challenge strain, only three animals were positive at 5 dpc, and the remainder by 9 dpc. In the VC group, the average titres against the vaccine strain were higher than against the challenge virus at 5 dpc, however from 9 dpc neutralising titres to the two viruses were comparable.

Two out of six UC sheep seroconverted to FMDV A/VIT/15/2012 with neutralising antibodies by 9 dpc and a further three by 14 dpc (Fig 4A). The neutralising antibody titres against the vaccine strain were lower overall in the UC sheep and sheep 15 did not reach a level considered positive against this virus at the time of euthanasia (12 dpc). Sheep 17 did not have FMDV neutralising antibodies up to 21 dpc. Overall there was a greater neutralising antibody response in the VC animals compared to the UC animals, in line with the expected results following priming of the immune system.

The median time to seroconversion to the structural proteins (determined by ELISA), as assessed by the Kaplan-Meier time to event analysis, was 1 day in the VC sheep and 12 days in the UC sheep (Fig 4B). This was a highly significant difference (p = 0. 0.00076).

The comparable time to seroconversion to the non-structural proteins (determined by ELISA) was 10 days in the VC sheep and 21 days in the UC sheep (Fig 4C), again a highly significant difference (p = 0.00278). However, it must be noted that UC sheep 17 had no signs of infection, four UC sheep were euthanized between 9 and 12 dpc, and after day 14, sampling was only done on a weekly basis, skewing the results.

## Discussion

Standardised vaccine potency studies quantify efficacy by level of protection from clinical disease. However, in the context of an outbreak, reduced susceptibility to infection and reduction in virus excretion is important to reduce transmission [21]. Previous studies have shown vaccination with high payload vaccines can achieve reduction in excretion, including with heterologous challenge, and with just 4 days between vaccination and challenge [11, 12, 22–27]. More often, as reported here, vaccination prevents clinical disease but is less effective at preventing sub-clinical infection and virus excretion [22, 24, 26, 27].

In this study, vaccination with an emergency high payload vaccine (>6PD_50_/dose) reduced clinical disease following heterologous challenge by direct contact just 4 dpv, but did not prevent sub-clinical infection or the occurrence of persistent infection. As all UC animals had to be euthanized by 21 dpc, their carrier status is unavailable for comparison. While there was a significant difference in the presence or absence of clinical disease and viraemia between the vaccinated and unvaccinated groups, there was no clear effect of vaccination on virus excretion. However, there were a number of factors that prevent any strong conclusions being drawn from these results. With direct-contact challenge, the challenge dose, and subsequently disease severity and virus excretion, is much lower than that seen with CB inoculation. The number of positive nasal and oral swab samples was small in both the UV and VC groups, and as the sheep were housed continuously with the donor sheep, we cannot discount positivity from environmental exposure rather than excretion in some cases. Additionally, sampling of the UC sheep was not comprehensive due to the requirement for early euthanasia. These inconclusive results are influenced by the animal to animal variation observed within each group and the small numbers of animals, which are common limitations when working with large animals in a BSL3 laboratory where space is often limited. Inherent variation between outbred animals is to be expected [22, 24, 26, 27].

There can also be differences in disease outcomes due to different animal species, virus serotype, or challenge method/severity [9, 11, 28, 29]. Coronary band inoculation is widely used in experiments using sheep, however it does not represent natural infection and in many cases results in a level of disease severity that is far beyond what sheep would experience in the field (rev by [30]). Notably, the observed clinical signs in the donor sheep were more severe than in the UC sheep. An important attribute of direct challenge methods like CB inoculation is reproducibility. All donors became infected and had similar immune responses, but there was still significant variation between individual donor sheep in clinical score and in virus excretion, particularly over the extended 9 day period, likely due to differences in individual animals’ ability to clear the infection.

This study has highlighted that sheep-to-sheep direct-contact challenge has several limitations in an experimental setting; primarily that the challenge dose and time the animal receives an infectious dose is unknown. Seroconversion times suggest the contact sheep were infected at different times over an eight day period, and one UC sheep (sheep 17) did not become infected. Variation in the amount of virus excreted by donors can influence exposure, although statistically there was no difference in total excreted virus between the replicate rooms in this study during the first 6 dpc, which is considered the period when the sheep are most infectious [20]. Pigs, which excrete larger volumes of virus, have also been used as donors, yet similar inconsistencies in challenge severity were observed [9, 31]. More recently intranasal instillation and aerosol masks have proven effective, reproducible challenge methods [29] Horsington *et al*., unpublished] that are more ‘natural’ than CB inoculation, and these approaches should be considered for future studies.

An important consideration for emergency vaccination is the time of challenge following vaccination. This significantly influences the level of protection, and it has been shown sterile protection is less common when challenge is just 4 dpv [9, 23, 25, 27, 31]. The onset of disease in the UC sheep, and time of seroconversion to NSP in all contact sheep, show infection time post-contact challenge varied, and indicate VC sheep 7 was infected very early (likely the first day of contact), whereas the remaining vaccinated sheep presumably had additional days for the development of a protective immune response before being exposed to an infectious dose. Virus neutralisation titre is generally considered a correlate of protection for FMDV in sheep, however protection in the absence of detectable neutralising antibodies has been reported [9, 11, 13, 32]. At the time of challenge, all VC sheep had either no detectable VN antibodies or VN titres considered negative. This may suggest a role of innate immunity in protection. A role for type I interferons in protection from FMDV has been shown in other species [33, 34], however in a similar study we found no association between serum interferon levels and protection in sheep (unpublished). A more detailed investigation into the relationship between innate immune responses to FMDV and protection in sheep would be beneficial, but was outside the scope of this study.

The difference in VN titres to the challenge and vaccine strains in the donor and UC sheep reflect the calculated r1 value for the virus against the vaccine. Similarly, disparate VN titres to the two strains were seen in the VC sheep at 5 dpc, with an anamnestic response from vaccination observed. This was reflected in the significant difference between the groups in the time to seroconversion to SP in ELISA. While r1 values are good indicators that a vaccine will work, they are less accurate at showing a vaccine won’t work, particularly when a high antigen payload is used [11, 35, 36]. Comparing genomic sequences is equally unreliable [37]. A better understanding of cell-mediated immune responses, or a combination of approaches is required to more accurately predict heterologous protection *in vitro*.

Foot-and-mouth disease continues to be a threat to farmers and livestock industries worldwide, especially in countries that currently enjoy trade advantages by being FMD-free. If an outbreak were to occur, the return to freedom must be as quick as possible, and emergency vaccination can play an important role in this. This report has shown that the A/VIT/15/2012 virus is pathogenic in sheep and, while vaccination with A22 IRQ did not provide sterile immunity or block persistent infection when sheep were challenged just 4 dpv, it was effective at reducing clinical disease and viraemia, supporting the use of this vaccine in an outbreak with A/VIT/15/2012 or similar viruses.

## Supporting information

S1 Fig Infection dynamics in donor sheep following CB inoculation. FMDV RNA detection in blood, nasal and oral swabs was performed using RT-qPCR, and is presented as log_10_ genome copy numbers/ml for blood or per swab. Clinical score is a cumulative index of FMD lesion distribution and clinical signs, where the maximum score is 10.
(PDF)

S1 Table Number of donor sheep positive for FMDV by RT-qPCR or VI in probang samples. (PDF)

## References

[1] McFadden AM, Tsedenkhuu P, Bold B, Purevsuren B, Bold D, Morris R. Epidemiology of the 2010 Outbreak of Foot-and-Mouth Disease in Mongolia. Transbound Emerg Dis. 2015;62(5):e45–51. doi: 10.1111/tbed.12208 . 24472307 10.1111/tbed.12208

[2] Ramanoon SZ, Robertson ID, Edwards J, Hassan L, Isa KM. Outbreaks of foot-and-mouth disease in Peninsular Malaysia from 2001 to 2007. Trop Anim Health Prod. 2013;45(2):373–7. doi: 10.1007/s11250-012-0226-x . 22826115 10.1007/s11250-012-0226-x

[3] Burrows R. The persistence of foot-and mouth disease virus in sheep. J Hyg (Lond). 1968;66(4):633–40. . 4303955 10.1017/s0022172400028369 PMC2130666

[4] Salt JS. The carrier state in foot and mouth disease—an immunological review. Br Vet J. 1993;149(3):207–23. doi: 10.1016/S0007-1935(05)80168-X . 8392891 10.1016/S0007-1935(05)80168-X

[5] Brooksby JB. Portraits of viruses: foot-and-mouth disease virus. Intervirology. 1982;18(1–2):1–23. doi: 10.1159/000149299 . 6288615 10.1159/000149299

[6] Cartwright B, Chapman WG, Sharpe RT. Stimulation by heterotypic antigens of foot-and-mouth disease virus antibodies in vaccinated cattle. Res Vet Sci. 1982;32(3):338–42. . 6179141

[7] Nagendrakumar SB, Srinivasan VA, Madhanmohan M, Yuvaraj S, Parida S, Di Nardo A, et al Evaluation of cross-protection between O1 Manisa and O1 Campos in cattle vaccinated with foot-and-mouth disease virus vaccine incorporating different payloads of inactivated O1 Manisa antigen. Vaccine. 2011;29(10):1906–12. doi: 10.1016/j.vaccine.2010.12.127 . 21236232 10.1016/j.vaccine.2010.12.127

[8] Vosloo W, Bastos AD, Sangare O, Hargreaves SK, Thomson GR. Review of the status and control of foot and mouth disease in sub-Saharan Africa. Rev Sci Tech. 2002;21(3):437–49. . 12523685 10.20506/rst.21.3.1349

[9] Cox SJ, Barnett PV, Dani P, Salt JS. Emergency vaccination of sheep against foot-and-mouth disease: protection against disease and reduction in contact transmission. Vaccine. 1999;17(15–16):1858–68. . 10217583 10.1016/s0264-410x(98)00486-1

[10] Doel TR, Williams L, Barnett PV. Emergency vaccination against foot-and-mouth disease: rate of development of immunity and its implications for the carrier state. Vaccine. 1994;12(7):592–600. . 8085375 10.1016/0264-410x(94)90262-3

[11] Horsington J, Zhang Z, Bittner H, Hole K, Singanallur NB, Alexandersen S, et al Early protection in sheep against intratypic heterologous challenge with serotype O foot-and-mouth disease virus using high-potency, emergency vaccine. Vaccine. 2015;33(3):422–9. doi: 10.1016/j.vaccine.2014.11.043 . 25483241 10.1016/j.vaccine.2014.11.043

[12] Parida S, Fleming L, Oh Y, Mahapatra M, Hamblin P, Gloster J, et al Emergency vaccination of sheep against foot-and-mouth disease: significance and detection of subsequent sub-clinical infection. Vaccine. 2008;26(27–28):3469–79. doi: 10.1016/j.vaccine.2008.04.026 . 18513836 10.1016/j.vaccine.2008.04.026

[13] Salt JS, Barnett PV, Dani P, Williams L. Emergency vaccination of pigs against foot-and-mouth disease: protection against disease and reduction in contact transmission. Vaccine. 1998;16(7):746–54. . 9562696 10.1016/s0264-410x(97)86180-4

[14] Knowles NJ, Samuel AR. Molecular epidemiology of foot-and-mouth disease virus. Virus Res. 2003;91(1):65–80. . 12527438 10.1016/s0168-1702(02)00260-5

[15] Mittal M, Tosh C, Hemadri D, Sanyal A, Bandyopadhyay SK. Phylogeny, genome evolution, and antigenic variability among endemic foot-and-mouth disease virus type A isolates from India. Arch Virol. 2005;150(5):911–28. doi: 10.1007/s00705-004-0469-6 . 15662482 10.1007/s00705-004-0469-6

[16] LaRocco M, Krug PW, Kramer E, Ahmed Z, Pacheco JM, Duque H, et al A continuous bovine kidney cell line constitutively expressing bovine alphavbeta6 integrin has increased susceptibility to foot-and-mouth disease virus. J Clin Microbiol. 2013;51(6):1714–20. doi: 10.1128/JCM.03370-12 ; PubMed Central PMCID: PMCPMC3716081. 23515553 10.1128/JCM.03370-12 PMC3716081

[17] LaRocco M, Krug PW, Kramer E, Ahmed Z, Pacheco JM, Duque H, et al Correction for LaRocco et al., A Continuous Bovine Kidney Cell Line Constitutively Expressing Bovine alphaVbeta6 Integrin Has Increased Susceptibility to Foot-and-Mouth Disease Virus. J Clin Microbiol. 2015;53(2):755 doi: 10.1128/JCM.03220-14 ; PubMed Central PMCID: PMCPMC4298512. 25617444 10.1128/JCM.03220-14 PMC4298512

[18] Moniwa M, Embury-Hyatt C, Zhang Z, Hole K, Clavijo A, Copps J, et al Experimental foot-and-mouth disease virus infection in white tailed deer. J Comp Pathol. 2012;147(2–3):330–42. doi: 10.1016/j.jcpa.2012.01.010 . 22520809 10.1016/j.jcpa.2012.01.010

[19] Mackay DK, Bulut AN, Rendle T, Davidson F, Ferris NP. A solid-phase competition ELISA for measuring antibody to foot-and-mouth disease virus. J Virol Methods. 2001;97(1–2):33–48. . 11483215 10.1016/s0166-0934(01)00333-0

[20] Alexandersen S, Zhang Z, Reid SM, Hutching G, Donaldson AI. Quantities of infectious virus and viral RNA recovered from sheep and cattle experimentally infected with foot-and-mouth disease virus O UK 20012002. Available from: http://www.fao.org/ag/againfo/commissions/docs/research_group/moen/App29.pdf. doi: 10.1099/0022-1317-83-8-1915 12124455 10.1099/0022-1317-83-8-1915

[21] Bravo de Rueda C, de Jong MC, Eble PL, Dekker A. Quantification of transmission of foot-and-mouth disease virus caused by an environment contaminated with secretions and excretions from infected calves. Vet Res. 2015;46:43 doi: 10.1186/s13567-015-0156-5 ; PubMed Central PMCID: PMCPMC4404111. 25928658 10.1186/s13567-015-0156-5 PMC4404111

[22] Barnett PV, Keel P, Reid S, Armstrong RM, Statham RJ, Voyce C, et al Evidence that high potency foot-and-mouth disease vaccine inhibits local virus replication and prevents the "carrier" state in sheep. Vaccine. 2004;22(9–10):1221–32. doi: 10.1016/j.vaccine.2003.09.024 . 15003651 10.1016/j.vaccine.2003.09.024

[23] Cox SJ, Parida S, Voyce C, Reid SM, Hamblin PA, Hutchings G, et al Further evaluation of higher potency vaccines for early protection of cattle against FMDV direct contact challenge. Vaccine. 2007;25(44):7687–95. doi: 10.1016/j.vaccine.2007.07.067 . 17913309 10.1016/j.vaccine.2007.07.067

[24] Cox SJ, Voyce C, Parida S, Reid SM, Hamblin PA, Hutchings G, et al Effect of emergency FMD vaccine antigen payload on protection, sub-clinical infection and persistence following direct contact challenge of cattle. Vaccine. 2006;24(16):3184–90. doi: 10.1016/j.vaccine.2006.01.037 . 16488060 10.1016/j.vaccine.2006.01.037

[25] Golde WT, Pacheco JM, Duque H, Doel T, Penfold B, Ferman GS, et al Vaccination against foot-and-mouth disease virus confers complete clinical protection in 7 days and partial protection in 4 days: Use in emergency outbreak response. Vaccine. 2005;23(50):5775–82. doi: 10.1016/j.vaccine.2005.07.043 . 16153756 10.1016/j.vaccine.2005.07.043

[26] Madhanmohan M, Nagendrakumar SB, Kumar R, Anilkumar J, Manikumar K, Yuvaraj S, et al Clinical protection, sub-clinical infection and persistence following vaccination with extinction payloads of O1 Manisa Foot-and-Mouth Disease monovalent vaccine and challenge in goats and comparison with sheep. Res Vet Sci. 2012;93(2):1050–9. doi: 10.1016/j.rvsc.2011.10.006 . 22079173 10.1016/j.rvsc.2011.10.006

[27] Madhanmohan M, Nagendrakumar SB, Srinivasan VA. Protection against direct in-contact challenge following foot-and-mouth disease vaccination in sheep and goats: the effect on virus excretion and carrier status. Vet Res Commun. 2010;34(3):285–99. doi: 10.1007/s11259-010-9353-x . 20352490 10.1007/s11259-010-9353-x

[28] Cox SJ, Barnett PV. Experimental evaluation of foot-and-mouth disease vaccines for emergency use in ruminants and pigs: a review. Vet Res. 2009;40(3):13 doi: 10.1051/vetres:2008051 ; PubMed Central PMCID: PMCPMC2695037. 19040829 10.1051/vetres:2008051 PMC2695037

[29] Stenfeldt C, Pacheco JM, Singanallur NB, Ferreira HC, Vosloo W, Rodriguez LL, et al Clinical and virological dynamics of a serotype O 2010 South East Asia lineage foot-and-mouth disease virus in sheep using natural and simulated natural inoculation and exposure systems. Vet Microbiol. 2015;178(1–2):50–60. doi: 10.1016/j.vetmic.2015.04.004 . 25937316 10.1016/j.vetmic.2015.04.004

[30] Alexandersen S, Zhang Z, Reid SM, Hutchings GH, Donaldson AI. Quantities of infectious virus and viral RNA recovered from sheep and cattle experimentally infected with foot-and-mouth disease virus O UK 2001. J Gen Virol. 2002;83(Pt 8):1915–23. doi: 10.1099/0022-1317-83-8-1915 . 12124455 10.1099/0022-1317-83-8-1915

[31] Aggarwal N, Zhang Z, Cox S, Statham R, Alexandersen S, Kitching RP, et al Experimental studies with foot-and-mouth disease virus, strain O, responsible for the 2001 epidemic in the United Kingdom. Vaccine. 2002;20(19–20):2508–15. . 12057606 10.1016/s0264-410x(02)00178-0

[32] Donaldson AI, Doel TR. Foot-and-mouth disease: the risk for Great Britain after 1992. Vet Rec. 1992;131(6):114–20. . 1326800 10.1136/vr.131.6.114

[33] Barnett PV, Cox SJ, Aggarwal N, Gerber H, McCullough KC. Further studies on the early protective responses of pigs following immunisation with high potency foot and mouth disease vaccine. Vaccine. 2002;20(25–26):3197–208. . 12163272 10.1016/s0264-410x(02)00242-6

[34] Dias CC, Moraes MP, Segundo FD, de los Santos T, Grubman MJ. Porcine type I interferon rapidly protects swine against challenge with multiple serotypes of foot-and-mouth disease virus. J Interferon Cytokine Res. 2011;31(2):227–36. doi: 10.1089/jir.2010.0055 . 20874428 10.1089/jir.2010.0055

[35] Eble PL, de Bruin MG, Bouma A, van Hemert-Kluitenberg F, Dekker A. Comparison of immune responses after intra-typic heterologous and homologous vaccination against foot-and-mouth disease virus infection in pigs. Vaccine. 2006;24(9):1274–81. doi: 10.1016/j.vaccine.2005.09.040 . 16289709 10.1016/j.vaccine.2005.09.040

[36] Nagendrakumar SB, Srinivasan VA, Madhanmohan M, Yuvaraj S, Parida S, Di Nardo A, et al Evaluation of cross-protection between O(1) Manisa and O(1) Campos in cattle vaccinated with foot-and-mouth disease virus vaccine incorporating different payloads of inactivated O(1) Manisa antigen. Vaccine. 2011. Epub 2011/01/18. doi: S0264-410X(11)00008-9 [pii] doi: 10.1016/j.vaccine.2010.12.127 . 21236232 10.1016/j.vaccine.2010.12.127

[37] Ludi AB, Horton DL, Li Y, Mahapatra M, King DP, Knowles NJ, et al Antigenic variation of foot-and-mouth disease virus serotype A. J Gen Virol. 2014;95(Pt 2):384–92. doi: 10.1099/vir.0.057521–0 . 24187014 10.1099/vir.0.057521-0 PMC7346513

