# Supplementary figures and images for: The protective capacity of high payload FMDV A22 IRQ vaccine in sheep against direct-contact challenge with a heterologous, contemporary FMDV A strain from South East Asia
Source: PLoS One. 2018 Jun 18;13(6):e0195302. doi: 10.1371/journal.pone.0195302 (PMC6005461; doi:10.1371/journal.pone.0195302)

Supplemental Figure S1

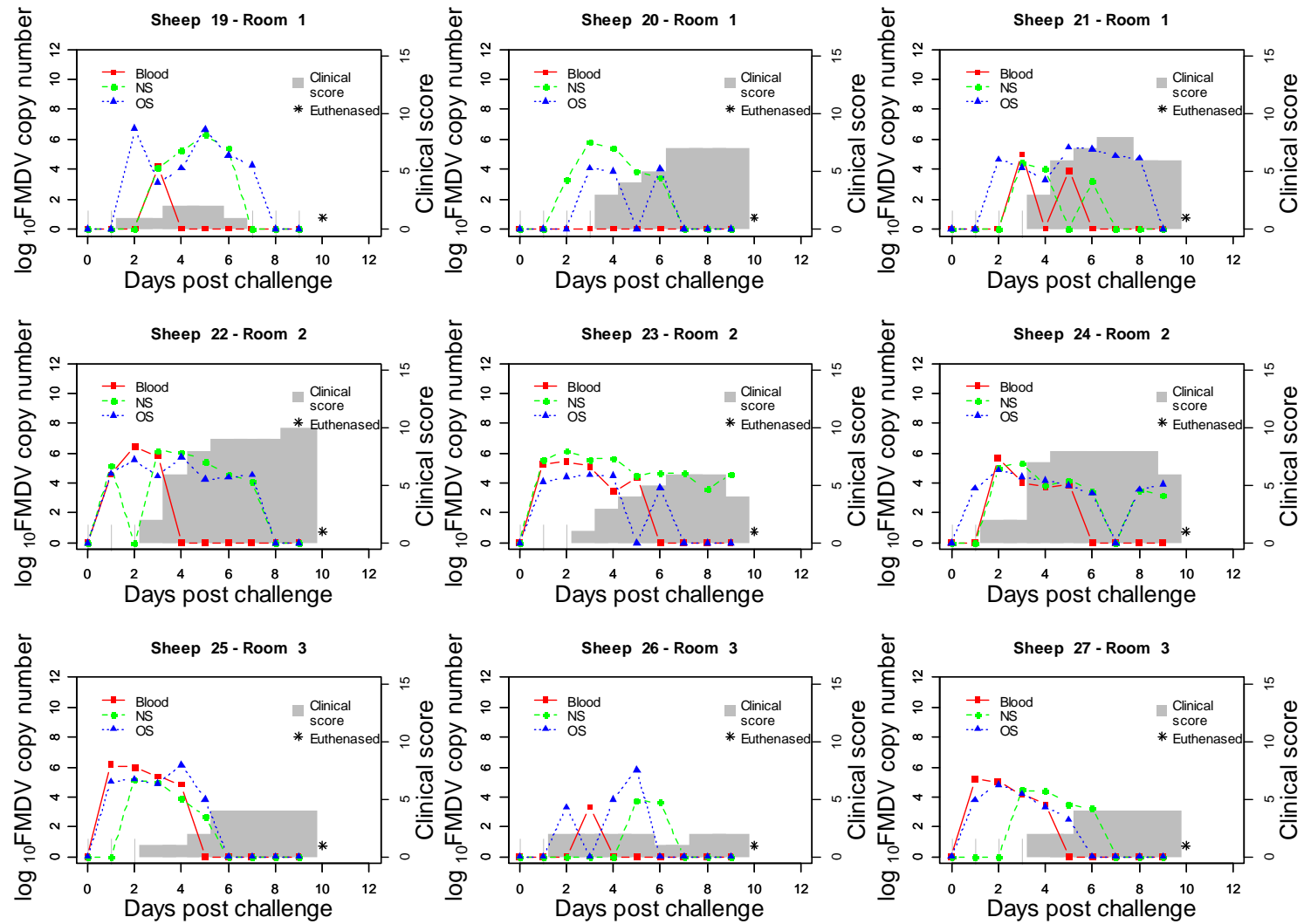

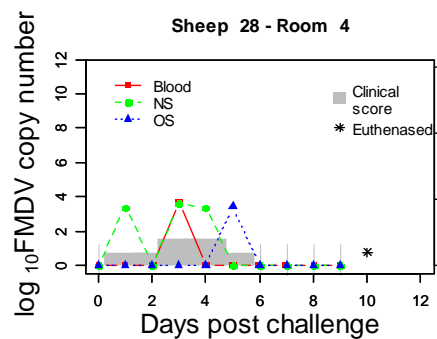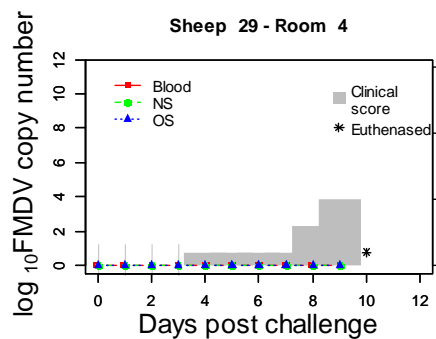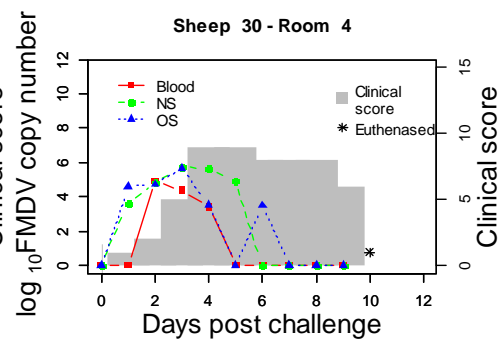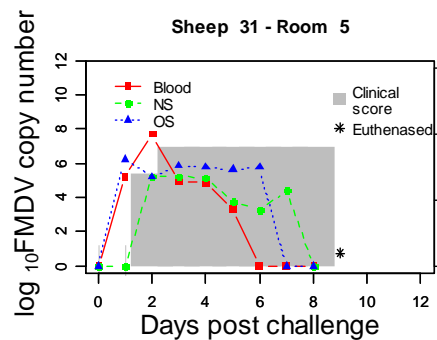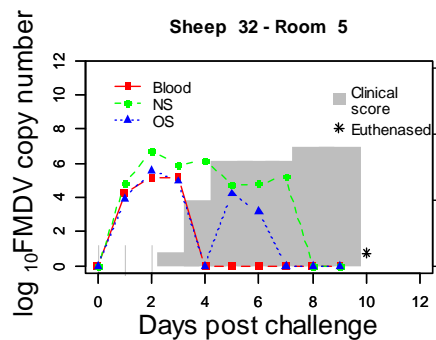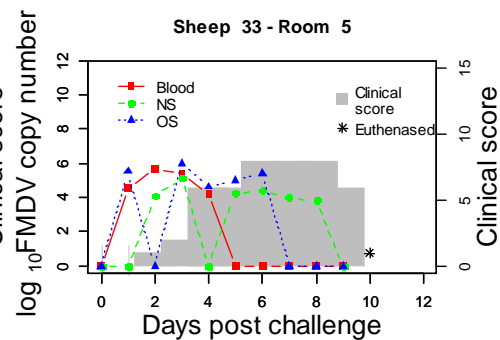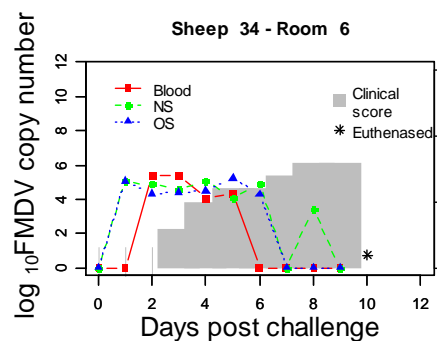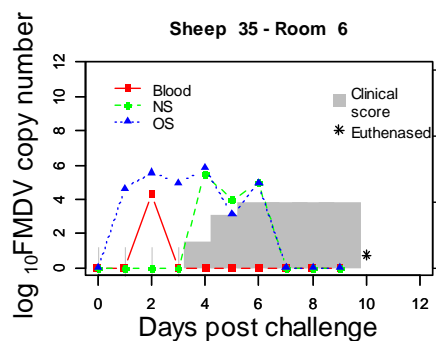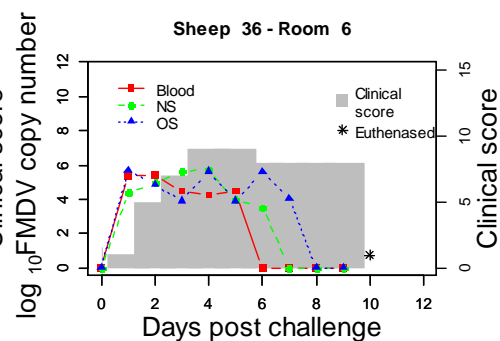

Supplement: S1 Fig — FMDV RNA detection in blood, nasal and oral swabs was performed using RT-qPCR, and is presented as log10 genome copy numbers/ml for blood or per swab. Clinical score is a cumulative index of FMD lesion distribution and clinical signs, where the maximum score is 10. (PDF) [file pone.0195302.s001.pdf]
