# Supplementary material for: The protective capacity of high payload FMDV A22 IRQ vaccine in sheep against direct-contact challenge with a heterologous, contemporary FMDV A strain from South East Asia
Source: PLoS One. 2018 Jun 18;13(6):e0195302. doi: 10.1371/journal.pone.0195302 (PMC6005461; doi:10.1371/journal.pone.0195302)
Supplement: S1 Table — (PDF) [file pone.0195302.s002.pdf]

S1 Table:

Sheep No.	Days Post-challenge					
	0		7		9	
	VI*	RT-qPCR	VI	RT-qPCR	VI	RT-qPCR
19	-	-	-	+	-	-
20	-	-	-	-	-	-
21	-	-	-	+	-	-
22	-	-	+	+	-	+
23	-	-	+	+	+	+
24	-	-	+	+	+	+
25	-	-	+	+	+	+
26	-	-	+	-	+	-
27	-	-	+	+	-	-
28	-	-	-	-	+	+
29	-	-	-	-	-	-
30	-	-	+	-	-	+
31	-	-	+	+	na	na
32	-	-	+	+	+	+
33	-	-	+	-	-	-
34	-	-	-	-	+	+
35	-	-	-	+	-	-
36	-	-	+	+	-	+

*VI = virus isolation on LFBK cells

+ = positive; - = negative; na = not available
